# Clinical Utility of Semistructured Interview and Scales to Assess Withdrawal Syndromes With Dose Reduction or Discontinuation of Selective Serotonin Reuptake Inhibitors or Serotonin Norepinephrine Reuptake Inhibitors

**DOI:** 10.1097/JCP.0000000000001491

**Published:** 2021-12-27

**Authors:** Fiammetta Cosci, Sara Romanazzo, Giovanni Mansueto, Petra Rontani, Michelle N. Levitan, Roseane D. Halkjœr-Lassen, Laiana A. Quagliato, Tomoyuki Nakamura, Ken Uematsu, Antonio E. Nardi, Misari Oe, Virginie-Anne Chouinard, Guy Chouinard

**Affiliations:** From the ∗ Department of Health Sciences; † Clinical Pharmacopsychology Laboratory, University of Florence, Florence, Italy; ‡ Department of Psychiatry and Neuropsychology, Maastricht University, Maastricht, the Netherlands; § Department of Experimental and Clinical Medicine, University of Florence, Florence, Italy; ∥ Laboratory of Panic and Respiration, Institute of Psychiatry of the Federal University of Rio de Janeiro, Rio de Janeiro, Brazil; ¶ Department of Neuropsychiatry, Kurume University School of Medicine, Kurume, Japan; # Psychotic Disorders Division, McLean Hospital, Harvard Medical School, Belmont, MA; ∗∗ Clinical Psychopharmacology and Therapeutics Unit; †† University Mental Health Institute of Montreal, Research Center Fernand Seguin, University of Montréal, Montréal, Québec, Canada.

**Keywords:** withdrawal syndrome, discontinuation, selective serotonin reuptake inhibitor, serotonin-noradrenaline reuptake inhibitor

## Abstract

**Background:**

Withdrawal syndromes can occur after dose reduction or discontinuation of selective serotonin reuptake inhibitors (SSRIs) or serotonin-norepinephrine reuptake inhibitors (SNRIs). Few measurement instruments are available to assess them: Diagnostic Clinical Interview for Drug Withdrawal 1–New Symptoms of SSRI and SNRI (DID-W1) and Discontinuation Emergent Signs and Symptoms (DESS) checklist. We assessed their interrater reliability, verified the percent agreement between the two, and tested DESS sensitivity and specificity on the basis of the diagnoses formulated via the DID-W1.

**Methods:**

One-hundred thirty-four subjects who referred for withdrawal at 3 outpatient facilities were enrolled and assessed via the DESS and the DID-W1. Percent agreement and Cohen κ were calculated to measure DID-W1 and DESS interrater reliability, as well as the agreement between DID-W1 and DESS items. Sensitivity and specificity of DESS were derived from the identification of true-positive, false-negative, true-negative, and false-positive on the DID-W1.

**Results:**

Both tools showed excellent interrater reliability (DID-W1 Cohen κ = 0.958; DESS Cohen κ = 0.81–1). The degree of agreement between DID-W1 and DESS items was poor or fair (Cohen κ < 0.40) for some items and moderate (Cohen κ = 0.41–0.60) for others. Sensitivity and specificity of DESS were 0.937 (true-positive = 60, false-negative = 4) and 0.285 (true-negative = 20, false-positive = 50), respectively.

**Conclusions:**

DID-W1 was a reliable method to identify and diagnose withdrawal syndromes. The DESS checklist showed to be a useful tool for detecting withdrawal SSRI/SNRI symptoms when the aim is to achieve high sensitivity to identify true positives.

Withdrawal is commonly seen with antidepressant use and has been reported in more than half (56%) of patients with discontinuation.^1^ In the first systematic review on selective serotonin reuptake inhibitors (SSRIs), Fava et al^2^ found up to 40% of patients experienced new-onset symptoms with abrupt discontinuation or gradual tapering of SSRIs. Fava et al^3^ also published the first systematic review on serotonin-norepinephrine reuptake inhibitors (SNRIs) showing that withdrawal symptoms occur after their abrupt discontinuation or gradual tapering.^3^

These reviews posited the need for a new classification of withdrawal symptoms^2,3^ to detect the range of symptoms experienced by patients and diagnose withdrawal syndromes specific to SSRI/SNRI.^4^ Based on classical concepts of central nervous system drug withdrawal, Chouinard and Chouinard^5^ proposed specific diagnostic criteria to identify 3 withdrawal syndromes, which can occur with decrease or discontinuation of SSRI/SNRI. These diagnostic criteria for SSRI/SNRI were elaborated from well-established withdrawal categories for central nervous system drugs. They include (1) new withdrawal symptoms, which are new to the patient and not part of the original psychiatric illness, as previously reported with benzodiazepines^6^; (2) rebound symptoms, which consist in a rapid return of the patient's original psychiatric illness at a greater intensity than before treatment; both first and second categories are transient, reversible, characterized by a peak of onset within 36 to 96 hours after discontinuation or dose reduction of SSRIs/SNRIs, and usually last up to 6 weeks^6^; and (3) persistent postwithdrawal disorders, which may consist of new withdrawal symptoms or rebound (preexisting) symptoms, but are long-lasting, severe, potentially irreversible, persisting more than 6 weeks and characterized by a peak of onset between 24 hours and 6 weeks.^7–9^

Recently, Cosci et al^10^ developed a semistructured diagnostic interview, the Diagnostic Clinical Interview for Drug Withdrawal 1–New Symptoms of SSRI and SNRI (DID-W1), to diagnose withdrawal syndromes at dose reduction or discontinuation of SSRI/SNRI according to Chouinard and Chouinard^5^ diagnostic criteria. In a pilot study, the DID-W1 interview showed excellent interrater agreement,^10^ allowing for the diagnosis of withdrawal syndromes. Another widely used tool to assess signs and symptoms associated with SSRI discontinuation is the Discontinuation Emergent Signs and Symptoms (DESS).^11^ It can be administered as a self-rated dimensional instrument or as a clinician-rated checklist. It attempts to parse out new symptoms, versus old symptoms, and whether the old symptoms are worse, unchanged, or improved. The tool has no diagnosis purposes and cannot be used to assess persistent postwithdrawal symptoms, which have been clearly identified years later the creation of the DESS and whose prevalence estimate is still in need of being measured.

The aim of the present study was to assess interrater reliability of the DID-W1 and the DESS, to verify the percent agreement between DID-W1 and DESS items, and to test DESS sensitivity and specificity using the DID-W1 as a diagnostic tool. There is need of differentiating withdrawal symptoms from syndromes and confront criteria with dimensional assessments.

## METHODS

### Participants

Participants were consecutively recruited at 3 third-level psychiatric outpatients' facilities (the Pharmachopsychology Service at the University of Florence, the Outpatient Resistant Depression Unit of Institute of Psychiatry at the Federal University of Rio de Janeiro, and the Kurume University Hospital) from July 2018 to December 2020 among patients for whom withdrawal symptomatology was suspected.

The following inclusion criteria were applied: (1) age 18 years or older; (2) history of SSRI/SNRI decrease/discontinuation; and (3) referring at least 1 symptom that occurred immediately after decrease or discontinuation of an SSRI or an SNRI. Exclusion criteria were (1) dose decrease/discontinuation of more than 1 SSRI/SNRI or decrease/discontinuation of SSRI/SNRI concurrent with dose decrease/discontinuation of other psychotropic medications, because their effects could overlap, and (2) intellectual disability.

Subjects were independently assessed by 2 raters per center using both the DID-W1^10^ and the DESS.^11^ Raters (ie, trained clinical psychologists or psychiatrists) had clinical experience and followed a join practical training with guidance of the authors of the interview and data collection for a previous pilot study.^10^ The raters did not communicate during the testing session or afterward regarding the assessment and the scoring. The filled DID-W1 and DESS were stored in sealed envelopes, which were opened at the time of statistical analysis. Sociodemographic and clinical data were collected as previously described.^12^

The study was approved by the Ethic Committee of the University Hospital Careggi (Florence, Italy), and participants provided written informed consent.

### Instruments

The DID-W1^10^ is a brief semistructured clinical interview that allows for the diagnosis of withdrawal syndromes with decrease or discontinuation of SSRI/SNRI according to Chouinard and Chouinard^5^ diagnostic criteria. The interview has 5 modules, previously described^10^: the DID-W1–Personal Data, collecting sociodemographic and clinical information (which allow also to discriminate between discontinuation-related symptoms and symptoms that are part of the original mental illness); the DID-W1–Screening Questions, screening on lifetime use of SSRI/SNRI; the DID-W1–Withdrawal Symptoms 1 (WS1), informing the diagnosis of current (section a) or lifetime (section b) new withdrawal symptoms; the DID-W1–Withdrawal Symptoms 2 (WS2), informing the diagnosis of current (section a) or lifetime (section b) rebound withdrawal symptoms; and the DID-W1–Withdrawal Symptoms 3 (WS3), informing the diagnosis of current (section a) or lifetime (section b) persistent postwithdrawal disorders. Items are rated with a dichotomous response format (yes/no). The administration of the interview requires on average 30 minutes. For the present study, the administration of the interview required a median of 35.5 minutes (minimum, 20 minute; maximum, 60 minutes).

In a preliminary study conducted in 17 subjects, the DID-W1 showed an excellent interrater agreement (% of agreement: 97.06%; Cohen κ = 0.85 with 95% confidence interval [CI] of 0.61–1.08; squared correlation coefficient = 0.72).^10^

The DESS^11^ is an instrument that queries for signs and symptoms associated with discontinuation of treatments inhibiting serotonin reuptake. A total score can be calculated, but subjects are considered at risk of experiencing a withdrawal syndrome if the number of DESS events increases by 4 or more during the discontinuation period. The instrument has 43 items and can be administered in a clinician-rated form (as in the present research), in a self-rated form, or as an interactive voice-response form. For the present research, subjects were asked whether they have experienced changes in symptoms since the last decrease or discontinuation of SSRI/SNRI and were invited to identify each symptom as new, old (but worse), old (but improved), old (but unchanged), or not present.^13,14^ Each item is scored 0/1.

### Statistical Analysis

Interrater reliability was tested via the percent agreement, Cohen κ,^15^ and 95% CI. Because κ cannot be directly interpreted, a squared correlation coefficient, named coefficient of determination, was used.^16^

The percent agreement was also used to verify the agreement between DID-W1-WS1a and DESS items. The following matching between DID-W1-WS1a and DESS items was applied: irritability (B.7: DID-W1-WS1a and 3: DESS); aggression (B.9: DID-W1-WS1a and 5: DESS); agitation (B.8: DID-W1-WS1a and 8: DESS); decreased concentration (B.5: DID-W1-WS1a and 10: DESS); cognitive symptoms (C.6: DID-W1-WS1a and 11: DESS); dysphoria (B.11: DID-W1-WS1a and 12: DESS); tremor (B.3: DID-W1-WS1a and 16: DESS); headaches (B.2: DID-W1-WS1a and 27: DESS); dizziness, lightheaded (C.2: DID-W1-WS1a and 29: DESS); sensory symptoms (C.5: DID-W1-WS1a and 40: DESS); anxiety (B.6: DID-W1-WS1a and items 1, 6, and 9: DESS); depression (B.10: DID-W1-WS1a and items 4 and 7: DESS); sleep disturbances (B.4: DID-W1-WS1a and items 13 and 14: DESS); general symptoms (C.1: DID-W1-WS1a and items 15, 21, 24, 30, 31, 32, and 33: DESS); neuromuscular symptoms (C.4: DID-W1-WS1a and items 17, 18, 19, 20, 25, and 26:DESS); nausea (B.1: DID-W1-WS1a and items 34 and 35: DESS); and gastrointestinal symptoms (C.3: DID-W1-WS1a and items 36, 37, and 38: DESS).

Strength of agreement, according to Landis and Koch,^17^ was slight or poor strength (Cohen κ = 0.0–0.2), fair (Cohen κ = 0.21–0.40), moderate (Cohen κ = 0.41–0.60), substantial (Cohen κ = 0.61–0.80), or almost perfect or perfect (Cohen κ = 0.81–1.0).

To calculate DESS sensitivity and specificity,^18^ subjects were split in 4 groups: true-positive (TP: ill and test positive); false-positive (FP: not ill but test positive); false-negative (FN: ill but test negative); and true-negative (TN: not ill and test negative)^19^ based on the assessment run via the DID-W1. Sensitivity (SN) was calculated as the proportion of TPs correctly identified: SN = TP/(TP + FN). Specificity (SP) was calculated as the proportion of TNs correctly identified: SP = TN/(TN + FP).^19,20^ Because, according to the DESS instructions, a change of 4 symptoms is needed to suspect a withdrawal syndrome,^11^ the rate of subjects who met a diagnosis of DID-W1 “current new withdrawal symptoms” was calculated among those who had at least 4 DESS “new symptoms” to test specificity and sensitivity of DESS regarding this diagnosis. Similarly, the rate of subjects who met a diagnosis of DID-W1 “current rebound withdrawal symptoms” was calculated among those who presented at least 4 DESS “old symptoms but worse” and, the rate of subjects who met the diagnosis of DID-W1 “current persistent postwithdrawal disorders” was calculated among those who presented at least 4 symptoms among DESS “new symptoms” and “old symptoms but worse.”

The statistical analyses were performed using IBM SPSS Statistics version 21. A *P* ≤ 0.05 was considered statistically significant.

## RESULTS

### Clinical Characteristics

Among the 150 subjects invited to participate, 134 took part in the research, 14 declined to participate—not being interested, and 2 did adhere for time reasons. Mean age ± SD was 39.63 ± 10.17 years, 59.7% (n = 80) were females. Approximately 29% (n = 39) had a high school education or higher, 40.3% (n = 54) were employed, and 47% (n = 63) were married. For the majority of subjects (83.6%; n = 112), the episode of possible withdrawal assessed for the present research was related to SSRI decrease/discontinuation. For 22 subjects (16.4%), the episode of possible withdrawal assessed was related to SNRI decrease/discontinuation.

### Interrater Reliability

DID-W1 showed excellent interrater reliability with a percent agreement of 97%, Cohen κ = 0.958 (SE = 0.021), 95% CI of 0.91–0.99, squared correlation coefficient of 0.918 (Table 1 shows detail for each DID-W1 module).

**TABLE 1:** Interrater Reliability of Each DID-W1 Diagnostic Module (n = 134)

Diagnostic Module	Percent Agreement	Cohen κ (SE)	95% CI	Coefficient of Determination
DID-W1-WS1a	100.0	1.000 (0.00)	—	1.000
DID-W1-WS1b	99.3	0.972 (0.028)	0.92–1.03	0.944
DID-W1-WS2a	99.3	0.974 (0.026)	0.92–1.02	0.948
DID-W1-WS2b	99.3	0.977 (0.023)	0.93–1.02	0.954
DID-W1-WS3a	99.3	0.983 (0.017)	0.94–1.02	0.966
DID-W1-WS3b	100.0	1.000 (0.00)	—	1.000

DID-W1-WS, Diagnostic Clinical Interview for Drug Withdrawal 1–New Symptoms of SSRI and SNRI, Withdrawal Symptoms.

DESS showed excellent interrater reliability, Cohen κ values ranging between 0.81 and 1 (Table 2).

**TABLE 2:** Interrater Reliability of DESS Items

Item	n	Percent Agreement	Cohen κ	95% CI	Coefficient of Determination
1. Anxiety	130	91.5	0.880	0.81–0.95	0.774
2. Elevated mood	130	96.2	0.820	0.67–0.97	0.672
3. Irritability	130	96.2	0.946	0.89–0.99	0.894
4. Worsening of mood	130	97.7	0.968	0.93–1.00	0.937
5. Anger attacks	130	96.9	0.942	0.88–0.99	0.887
6. Panic or anxiety attacks	130	95.4	0.940	0.89–0.98	0.884
7. Bouts of crying	130	98.5	0.977	0.94–1.01	0.954
8. Agitation	130	96.9	0.958	0.92–0.99	0.917
9. Feeling unreal	130	98.5	0.975	0.94–1.01	0.950
10. Trouble concentrating	130	96.2	0.947	0.90–0.99	0.897
11. Problems with memory	130	98.5	0.978	0.95–1.01	0.956
12. Mood swings	130	94.6	0.920	0.86–0.97	0.864
13. Insomnia	130	97.7	0.970	0.94–1.00	0.941
14. Nightmares	130	97.7	0.963	0.92–1.00	0.927
15. Sweating	130	95.4	0.922	0.86–0.98	0.850
16. Shaking	130	96.2	0.935	0.88–0.99	0.874
17. Muscle tension	130	99.2	0.988	0.96–1.01	0.976
18. Muscle aches	130	98.5	0.973	0.93–1.01	0.947
19. Restless feeling in legs	130	97.7	0.955	0.91–0.004	0.912
20. Muscle cramps	130	96.9	0.933	0.87–0.99	0.870
21. Fatigue	130	96.2	0.950	0.91–0.99	0.902
22. Unsteady gait	130	97.7	0.944	0.88–1.01	0.891
23. Blurred vision	130	98.5	0.963	0.91–1.01	0.927
24. Sore eyes	130	96.2	0.894	0.80–0.98	0.799
25. Uncontrollable mouth/tongue movements	130	98.5	0.883	0.72–1.04	0.779
26. Problems with speech	130	99.2	0.975	0.93–1.02	0.950
27. Headache	130	96.9	0.959	0.92–0.99	0.919
28. Increased saliva	130	96.9	0.828	0.67–0.98	0.685
29. Dizziness, vertigo	129	98.4	0.979	0.95–1.01	0.958
30. Nose running	130	97.7	0.903	0.79–1.01	0.815
31. Shortness of breath	130	98.5	0.977	0.94–1.01	0.954
32. Chills	130	97.7	0.954	0.90–1.00	0.910
33. Fever	130	96.9	0.891	0.79–0.99	0.793
34. Vomiting	130	96.9	0.885	0.77–0.99	0.783
35. Nausea	130	98.5	0.979	0.95–1.01	0.958
36. Diarrhea	130	97.7	0.957	0.91–1.00	0.916
37. Stomach cramps	129	96.9	0.931	0.87–0.99	0.866
38. Stomach bloating	130	95.4	0.908	0.84–0.98	0.824
39. Unusual visual sensation	130	100.0	1.000	—	1.000
40. Burning, numbness	130	99.2	0.985	0.95–1.01	0.970
41. Unusual sensitivity to sound	130	98.5	0.967	0.92–1.01	0.935
42. Ringing in the ears	130	99.2	0.983	0.95–1.01	0.966
43. Unusual tastes or smells	130	99.2	0.974	0.92–1.02	0.948

### Percent Agreement Between DID-W1-WS1a and DESS

Items with a slight or poor strength of agreement were as follows: anxiety, depression, anger or aggression, agitation, cognitive symptoms, dysphoria, sleep disturbances, headache. Irritability general symptoms, neuromuscular symptoms, and nausea showed a fair strength of agreement. Decreased concentration, tremor, dizziness or lightheaded, gastrointestinal symptoms, and sensory symptoms had a moderate strength of agreement (Table 3).

**TABLE 3:** Percent Agreement Between DID-W1–Withdrawal Symptoms 1a Items and DESS Items

Item	n	Percent Agreement	Cohen κ	95% CI	Coefficient of Determination
Anxiety	82	46.3	0.147	0.06–0.24	0.021
Irritability	81	71.6	0.397	0.21–0.58	0.157
Depression	81	55.6	0.143	−0.05 to 0.33	0.020
Anger/aggression	81	74.1	0.120	−0.12 to 0.36	0.014
Agitation	81	71.6	0.162	−0.07 to 0.40	0.026
Decreased concentration	82	69.5	0.414	0.25–0.58	0.171
Cognitive symptoms	80	51.3	0.179	0.06–0.29	0.032
Dysphoria	81	61.7	0.162	0.007–0.32	0.026
Sleep disturbances	82	53.7	0.146	−0.02 to 0.31	0.021
General symptoms	80	67.5	0.348	0.14–0.55	0.121
Tremor	82	79.3	0.504	0.31–0.70	0.254
Neuromuscular symptoms	80	71.3	0.391	0.19–0.60	0.153
Headache	82	51.2	0.148	0.02–0.27	0.021
Dizziness/lightheaded	80	76.3	0.517	0.33–0.70	0.267
Nausea	82	63.4	0.317	0.15–0.48	0.100
Gastrointestinal symptoms	80	83.8	0.541	0.36–0.72	0.292
Sensory symptoms	80	80.0	0.512	0.32–0.71	0.262

### Sensitivity and Specificity of DESS

Sensitivity and specificity of DESS were 0.937 (93.7%; TP = 60, FN = 4) and 0.285 (28.5%; TN = 20, FP = 50), respectively. The ability of DESS to correctly identify TPs with a DID-W1-WS1a diagnosis of “current new withdrawal symptoms” was low (sensitivity of 0.087 [8.7%; TP = 2, FN = 21]), whereas the ability to correctly identify TNs was high (specificity of 0.900 [90%; TN = 100, FP = 11]). Similar results were found for the diagnostic module DID-W1-WS2a, which pertains to “current rebound withdrawal symptoms” (sensitivity: 0.083 [8.3%; TP = 2, FN = 22]; specificity: 0.918 [91.8%; TN = 101, FP = 9]). As what concerns the DID-W1-WS3a diagnosis of “current persistent postwithdrawal disorders,” DESS sensitivity and specificity were 0.931 (93.1%; TP = 41, FN = 3) and 0.500 (50%; TN = 45, FP = 45), respectively.

## DISCUSSION

The DID-W1 showed excellent interrater reliability (percent agreement of 97%), which is consistent with the preliminary study.^10^ The DESS checklist also showed excellent interrater reliability.

The degree of agreement between DID-W1-WS1a and DESS differed according to items. A poor strength of agreement was found for psychological symptoms (ie, anxiety, depression, anger or aggression, agitation, cognitive symptoms, dysphoria, sleep disturbance), whereas a moderate strength of agreement (Cohen κ value = 0.41–0.60) was found for physical symptoms (ie, tremor, dizziness or lightheaded, gastrointestinal symptoms, sensory symptoms). It can be hypothesized that a checklist, such as the DESS, may more readily identify physical symptoms, whereas psychological symptoms may be more fully captured using tailored questions in a semistructured interview, such as the DID-W1.

DESS sensitivity and specificity were inversely proportional as expected^21^ and suggested that the DESS should be preferred to identify TPs, thus as a screening tool. However, when the 3 withdrawal syndromes were considered, sensitivity was low, and specificity was high for current new withdrawal symptoms and current rebound withdrawal symptoms, and vice versa for current persistent postwithdrawal disorders. Thus, apparently, the DESS is a good screening tool when the interest is focused on current persistent postwithdrawal disorders. The results can be explained by the low rate of the diagnoses current new withdrawal symptoms and current rebound withdrawal symptoms (2 subjects per diagnosis in the whole sample) if compared with the diagnosis of persistent postwithdrawal disorders, which was observed in 41 subjects over 134.

One limitation is the relatively small sample size, which does not allow for higher rates of current new withdrawal symptoms and current rebound withdrawal symptoms diagnoses and also introduces a limitation related to the questioned independence of the sensitivity/specificity and prevalence. Many empirical studies suggested that the disease prevalence may impact sensitivity and specificity. However, this sample of 134 subjects is unique in the literature on this topic, which has been largely explored via Web reports. Another limitation is that individuals were retrospectively assessed. Future prospective studies are warranted. The population under observation is a strength of the study. In addition, this is the first research examining interrater reliability of DESS and DID-W1, strength of agreement between their items, DESS sensitivity, and specificity.^22^

The present study has possible implications. The first area of application of DESS and DID-W1 is in naturalistic studies aimed at identifying prevalence of withdrawal syndromes; such prevalence is still unknown in the literature. Longitudinal studies may also benefit from the use of these instruments to clarify the relationships between withdrawal syndromes and other manifestations of drug-induced behavioral changes (eg, refractoriness, loss of effects).^23^ The second area has to do with the differentiation between relapse and withdrawal in trials aimed at testing for both short-term effects and long-term, preventive, or prophylactic effects of psychotropic medications.^24–26^ The various effects of discontinuing treatment with psychotropic medications can compromise the scientific soundness of research, especially in trials, which involve discontinuing a previous or current active treatment to a placebo. They are in need of distinguishing between early withdrawal-associated reactions (eg, new withdrawal symptoms and rebound), late withdrawal-associated reactions (eg, persistent postwithdrawal disorders), and later, posttreatment discontinuation reemergence of psychiatric illness. The third area of potential application is the identification of treatment resistance depression, discriminating it from tolerance phenomena within the heterogeneous components of the category.^27^

In addition, being the DESS is a good screening tool for current persistent postwithdrawal disorders, it might be routinely used by psychiatrists and clinical psychologists for the assessment of patients undergoing SSRI/SNRI discontinuation; the fact that the DESS is generally proposed as a self-rating scale reinforces its use as a screening method. In case the DESS suggests a possible withdrawal syndrome, and if the clinician can devote approximately 30 minutes to further assess the patient, a more refined clinimetric diagnosis^28^ might be reached via the DID-W1. Particularly persistent postwithdrawal disorders might need a more accurate assessment representing a major clinical problem. Differently from new withdrawal and rebound symptoms (which are de facto acute withdrawal), they are long lasting, severe, and reduce the working, social, and family functioning of the patients.^29^ In addition, the use of the DIDW-1 might help clinicians in differentiating between withdrawal and relapse. This is another vexing clinical dilemma with serious implications because a diagnostic mistake may drive clinicians to inadequately reintroduce the SSRI/SNRI. In practice, having assessed the patients via the DID-W1 and a diagnostic interview for *DSM* (*Diagnostic and Statistical Manual of Mental Disorders*) psychiatric disorders, clinicians may use the DID-W1 diagnoses as exclusion criteria for *DSM* diagnoses similarly to what they do for medical conditions. The overall priority, thus, would be to formulate an accurate diagnosis, which is the condition sine qua non for an appropriate treatment and for prevention.^29^ Further, the use of DESS as a screening tool and DID-W1 as a diagnostic tool follows the principles of incremental validity, which refers to the unique contribution or incremental increase in predictive power associated with the inclusion of a particular assessment procedure in the clinical decision process.^30^ Although the DESS is focused on current symptoms, the DID-W1 explores both current and lifetime clinical phenomena and provides a chronological appraisal of symptoms as well as a longitudinal development of disorders.^31^

In brief, the present research suggests that the DID-W1 is a reliable tool to diagnose withdrawal syndromes after dose decrease or discontinuation of SSRI/SNRI according to Chouinard and Chouinard^5^ criteria, and the DESS is an adequate screening tool for withdrawal syndromes, especially persistent postwithdrawal disorders. Because these instruments are available, and their properties are known, we recommend using them in clinical and research settings to refine the use and indications of antidepressants, including differences in drug-induced behavioral changes among antidepressants.^4^
